# Measuring progress towards universal health coverage: national and subnational analysis in Ethiopia

**DOI:** 10.1136/bmjgh-2019-001843

**Published:** 2019-11-01

**Authors:** Getachew Teshome Eregata, Alemayehu Hailu, Solomon Tessema Memirie, Ole Frithjof Norheim

**Affiliations:** 1 Department of Global Public Health and Primary Care, University of Bergen, Bergen, Norway; 2 Ministry of Health of Ethiopia, Addis Ababa, Ethiopia; 3 Department of Paediatrics and Child Health, College of Health Sciences, Addis Ababa University, Addis Ababa, Ethiopia; 4 Department of Global Health and Population, Harvard University T H Chan School of Public Health, Boston, Massachusetts, USA

**Keywords:** health policies and all other topics, health economics, health services research, health systems evaluation, health systems

## Abstract

**Introduction:**

Aiming for universal health coverage (UHC) as a country-level goal requires that progress is measured and tracked over time. However, few national and subnational studies monitor UHC in low-income countries and there is none for Ethiopia. This study aimed to estimate the 2015 national and subnational UHC service coverage status for Ethiopia.

**Methods:**

The UHC service coverage index was constructed from the geometric means of component indicators: first, within each of four major categories and then across all components to obtain the final summary index. Also, we estimated the subnational level UHC service coverage. We used a variety of surveys data and routinely collected administrative data.

**Results:**

Nationally, the overall Ethiopian UHC service coverage for the year 2015 was 34.3%, ranging from 52.2% in the Addis Ababa city administration to 10% in the Afar region. The coverage for non-communicable diseases, reproductive, maternal, neonatal and child health and infectious diseases were 35%, 37.5% and 52.8%, respectively. The national UHC service capacity and access coverage was only 20% with large variations across regions, ranging from 3.7% in the Somali region to 41.1% in the Harari region.

**Conclusion:**

The 2015 overall UHC service coverage for Ethiopia was low compared with most of the other countries in the region. Also, there was a substantial variation among regions. Therefore, Ethiopia should rapidly scale up promotive, preventive and curative health services through increasing investment in primary healthcare if Ethiopia aims to reach the UHC service coverage goals. Also, policymakers at the regional and federal levels should take corrective measures to narrow the gap across regions, such as redistribution of the health workforce, increase resources allocated to health and provide focused technical and financial support to low-performing regions.

**Key questions**

**What is already known?**

- Measurement of service coverage is important for monitoring progress towards universal health coverage (UHC).
- The WHO and the World Bank has made country estimates of UHC service coverage, including Ethiopia.

**What are the new findings?**

- This paper estimates UHC service coverage for Ethiopia for the year 2015.
- The estimated subnational UHC service coverage varies substantially across regions and programme areas.

**What do the new findings imply?**

- Ethiopia should make an extra effort to achieve UHC service coverage goals in the next decade.
- Policymakers in Ethiopia should be cognisant of the subnational variation in UHC service coverage and should take corrective measures to narrow the gap across regions.

## Introduction

Universal health coverage (UHC) is realised when everyone has access to quality essential healthcare services with financial risk protection.^1^ The United Nations General Assembly, as part of the Sustainable Development Goal for health, calls on all countries to ensure UHC by 2030.^2^ Health services that should be provided include essential promotive, preventive, curative, rehabilitative and palliative health services.^1^ However, each year, almost half of the world’s population cannot access needed health services and about 100 million people are forced into extreme poverty because of health expenses. Globally, about 800 million people experience catastrophic financial hardship due to out-of-pocket healthcare spending (ie, spending more than 10% of their total income for healthcare).^3^

Ethiopia is one of the countries with a substantial disease burden from reproductive, maternal, neonatal and child health (RMNCH) and infectious diseases.^4^ Recent evidence also shows that the magnitude of non-communicable disease (NCD) and injuries is rising in Ethiopia.^5^ In addition, coverage of both basic health services and health service utilisation is low, and there is a high geographical (urban-rural area and regional/subnational) inequality in service coverage.^6^ Furthermore, catastrophic out-of-pocket spending is high.^7^

Cognisant of these challenges, the government of Ethiopia has developed key strategies to lead to UHC. These strategies include the following: first, defining the Essential Health Service Package of the country and identifying prioritised health interventions; second, exemption or cost-sharing of high-priority interventions; third, expansion of community-based health insurance programmes; fourth, integration of health services within other sectors from the national to the district level to address social determinants of health and fifth, establish emergency preparedness and response units at the level of both the national and Regional Health Bureaus (RHBs). Taken together, these strategies can improve the coverage of essential health services, reduce inequalities and provide financial risk protection.^8 9^

Despite a promise to provide all needed service to the whole population, UHC must be realised progressively, especially in resource-constrained countries. Responsibilities in the provision of UHC need to be redefined to a comprehensive set of essential health services that the government can guarantee to the entire population.^10^ Therefore, with UHC as a global and country-level health policy goal, the need to measure and track its progress over time is imperative. WHO and World Bank (WB) have jointly identified two indicators for monitoring progress towards UHC: essential health service coverage (target number 3.8.1) and financial risk protection (target number 3.8.2). The 2030 UHC-Sustainable DevelopmentGoals (SDG) targets are intended to reach at least 80% for service coverage and 100% for financial risk protection.^11^

One challenge in the identification of UHC monitoring indicators is to decide how comprehensive the indicators should be to represent all essential health services in the system and control the number of indicators to enable comparability across countries that differ in terms of epidemiological and demographic characteristics. Boerma *et al* argue that country-level monitoring of UHC should address the epidemiological and demographic peculiarities in the country, while the inter-country comparison is more important for monitoring and comparing progress towards UHC.^12^

Hogan *et al* have proposed a metric for the monitoring of UHC: in particular, target 3.8.1. This seminal paper constructs a composite indicator to estimate UHC in a single number.^3^ The indicator comprises four elements: reproductive, maternal, new-born and child health; infectious diseases; NCDs and service capacity and access. The authors argue that the application of this single indicator can be used to compare the progress of different countries and monitor a country’s progress towards UHC.^3^

Although Wagstaff and colleagues were able to demonstrate the feasibility of an index-based approach to measuring, monitoring and comparing progress towards UHC,^13^ only a few empirical studies attempt to estimate UHC status at the country level. Barasa, Nguhiu and McIntyre use Demographic and Health Survey (DHS) data to estimate Kenya’s UHC progress using tracer indicators grouped as preventive and treatment interventions.^14^ In Ethiopia, a case study regarding the identification of a UHC monitoring tool recommends that the UHC tracer indicators should be comprehensive, few in number and focused on impact, outcome and health systems dimension indicators. The case study also supports the inclusion of indicators that can capture NCDs.^15^ Except for Hogan *et al*,^3^ we have not found other empirical studies estimating the UHC status for Ethiopia, and none have attempted to demonstrate subnational variations in universal service coverage. In addition, the Ministry of Health does not use an explicitly defined UHC monitoring framework. Therefore, this study aimed to estimate the 2015 national and subnational UHC status for Ethiopia, which could serve as a baseline to monitor Ethiopia’s progress towards UHC.

## Methods

### The Ethiopian health system

Ethiopia is the second most populous country in Africa, with a total population of about 105 million in 2016.^16^ Administratively, Ethiopia is divided into nine regional states (Tigray, Afar, Amhara, Oromia, BenshangulGumuze, Southern Nations, and National Region (SNNPR), Somali and Harari) and two chartered cities (Addis Ababa and Dire Dawa).

Healthcare delivery in Ethiopia is organised in a three-tier system.^8^ The first, at the district level, is the primary healthcare unit (PHCU). The PHCU comprises one primary hospital, which can serve a population of about 60 000–100 000; four health centres (each serving a population of 15 000–25 000) and five health posts are attached to each health centre (each health post serving 3000–5000 people). The second level comprises general hospitals, each serving a population of 1–1.5 million, while the third level comprises specialised hospitals for a population of 3.5–5 million.

While the Federal Ministry of Health is responsible for the formulation and harmonisation of health programmes and strategies, the RHBs are mostly responsible for actual implementations. The budget flows to RHBs in two ways. From one side, the RHBs receive about 5%–10% of the total annual regional budget. This part of the budget is mainly spent on salaries for health professionals, procurement of medical supplies and procurement of drugs. The regions also use this part of the budget for construction and expansion of health centres and primary hospitals. The RHBs have a full mandate on this part of the budget. On the other side, RHBs receive an additional earmarked budget for specific programmes from external sources via the Federal Ministry of Health. In addition, the Ministry of Health also distributes un-earmarked funds from the SDG pool fund.

### Service coverage indicators

In this study, we applied the approaches described by Hogan *et al* and the WHO/WB report on tracking progress towards UHC, with some modifications.^3 11^ We used local data sources and checked that the indicators were also relevant for Ethiopia and that the data were available for all nine regions and the two city administrations. The selected indicators were well aligned with Ethiopia’s priorities, set by the health sector transformation plan.^8^ Sixteen indicators are from four major categories: RMNCH, infectious diseases, NCDs and service capacity and access. Tracer indicators in the area of RMNCH were as follows: family planning (FP) demand satisfied with a modern method among married women or in a union; pregnancy care (PC); immunisation for infants with three doses of pentavalent vaccine and care-seeking for children with suspected pneumonia. For infectious diseases, tracer indicators were tuberculosis treatment coverage (TB cases detected and cured); HIV treatment coverage; use of insecticide-treated bed nets among populations at risk of malaria and household access to at least basic sanitation services. For NCDs, we used the following: prevalence of non-raised blood pressure (BP), mean fasting plasma glucose, cervical cancer screening and prevalence of tobacco non-smoking. To assess service capacity and access, we used hospital bed density, health worker density, access to essential medicines and the International Health Regulations core capacity index.

### Data sources

We used a variety of data sources from Ethiopia (table 1), namely Ethiopia’s Health Management Information System (HMIS),^17^ Ethiopia’s 2016 DHS (EDHS),^18^ the 2015 Malaria Indicator Survey (MIS),^19^ the NCD STEPwise approach to noncommunicable disease risk factor surveillance (STEPS) survey,^20^ the 2016 Service Readiness and Availability (SARA) survey^21^ and a Human Resource Information System (HRIS). Also, health security (HS) information was collected from administrative records at the Federal Ministry of Health and regional health offices. Since this indicator is only available at national level only, it is excluded from subnational analysis.

**Table 1:** Sources of data and indicator description for the UHC service coverage tracer indicators

Major indicators	Tracer indicator	Description	Data source
RMNCH	Family planning	Demand satisfied with modern methods among women 15–49 who are married or in a union	EDHS
	Pregnancy care	Average coverage of 4 or more antenatal visits and skilled birth attendants	EDHS
	Full child immunisation	One-year-old children who have received 3 doses of vaccine containing diphtheria, tetanus and pertussis	EDHS
	Child treatment	Care-seeking behaviour for children with suspected pneumonia	EDHS
Infectious diseases	Tuberculosis treatment	TB cases detected and cured	WHO
	HIV treatment	People living with HIV and receiving antiretrovirals (ART)	HMIS
	Malaria prevention	The population at risk sleeping under insecticide-treated bed nets	MIS
	Improved water and sanitation	Average coverage of households with access to improved water and sanitation	EDHS
NCDs	Prevention of CVD	Prevalence of no raised blood pressure	STEPS
	Management of DM	Prevalence of no raised blood glucose	STEPS
	Cervical cancer screening	Cervical cancer screening among women 30–49	STEPS
	Tobacco control	Adults age ≥15 years not smoking tobacco in the last 30 days	STEPS
Service capacity and access	Hospital access	In-patient admissions per capita	HMIS
	Health worker density	Health professionals per capita physicians, psychiatrists and surgeons	HRIS
	Access to essential medicines	The average proportion of the WHO-recommended core list of essential medicines present in health facilities	SARA
	Health security	International Health Regulations core capacity index	Primary

CVD, cardiovascular disease; DM, diabetes mellitus; EDHS, Ethiopia’s Demographic and Health Survey; HMIS, Health Management Information System; HRIS, Human Resource Information System; MIS, Malaria Indicator Survey; NCDs, non-communicable diseases; RMNCH, reproductive, maternal, neonatal and child health; SARA, Service Readiness and Availability; TB, tuberculosis; UHC, universal health coverage.

The Ethiopian 2016 DHS data were used to estimate UHC service coverage for RMNCH indicators. For measurement of the malaria prevention indicator, the 2015 Ethiopian MIS survey was used. The 2016 SARA survey was used to estimate the coverage of essential medicine. The SARA survey generates a set of core indicators on key inputs and outputs of the health scheme, which can be applied to assess progress in the health system, strengthening over time.^22^ To estimate service coverage for the prevention of cardiovascular disease (CVD), management of diabetes mellitus (DM), cervical cancer screening and tobacco control, the NCD STEPS survey was applied. The Ethiopia STEPS are a nationally representative survey to gather comprehensive data on risk factors for NCDs, injuries and violence in Ethiopia. To estimate HIV treatment coverage, HMIS data, which is routinely collected from service provision at each facility, was used. The data source for health workforce (HWF) density are the HRIS of the Ministry of Health.

### Data analysis

### Estimation of the UHC service coverage index

The UHC coverage index was constructed from geometric means of the four major component indicators.^3^ For the RMNCH category, the geometric mean of FP, PC, immunisation and child healthcare (CHC) were taken; for FP, contraceptive prevalence rate; for PC, a combination of prevalence of births attended by a skill birth attendant and prevalence of antenatal care coverage (ANC4+); for immunisation, DPT3 coverage and for CHC, treatment for childhood pneumonia in the last 2 weeks were used as follows:

RMNCH =${\left(FP*PC*DPT3*CHC\right)}^{1/4}$

For measurement of UHC service coverage in the infectious disease category, tuberculosis treatment (TB) was measured using the TB case detection rate and cure rate; antiretroviral treatment (ART) coverage was measured using people living with HIV who are currently on ART; water and sanitation (WASH) was measured using the average coverage of households with access to improved water and sanitation and Long-lasting insecticidal nets (LLIN) coverage was used.

Infectious = ($TB*ART*WASH*ITNright{)}^{1/4}$

LLIN coverage was not accounted for in Addis Ababa since the area is malaria-free.

NCD service coverage was calculated by a geometric mean of non-raised BP, fasting blood plasma glucose level (FPG), cervical cancer screening coverage and prevalence of non-tobacco users. We used the 2015 STEPS survey to compute the four tracer indicators in this category. The non-raised BP rate was measured by a prevalence of systolic BP<140 mm Hg or diastolic BP<90 mm Hg among adults aged 18 years and older. The FPG rate was measured by a prevalence of fasting plasma glucose of ≥7.0 mmol/L or those on medication for raised blood glucose among adults aged 18 years or older. The cervical cancer screening rate was measured by a proportion of women aged 30–49 years who reported ever having had a screening test for cervical cancer using any of the methods (visual inspection with aceticaccede, pap smear and human papillomavirus test). For measurement of no tobacco use, the proportion of adults 15 years and older who have not smoked tobacco in the last 30 days of survey time was applied:

$NCD=(BP*FPG*CancerScreening*Tobaccoright{)}^{1/4}$

We used the prevalence of non-raised BP to estimate the service coverage for ‘prevention of CVD’ and the prevalence of non-raised blood glucose to estimate the service coverage for ‘management of DM’. Since these two indicators are not measured in a proportion, we used the rescaling formulas recommended by WHO/WB:^3^

- Rescaled value for non-raised BP = (x – 50)/(100 – 50)*100, where x is the non-raised BP.
- Rescaled value for the management of non-FPG = (7.1 – y) / (7.1 – 5.1) *100, where y is the mean fasting plasma glucose.

For measurement of health service capacity and access (HSCA) coverage, hospital access (HP), HWF density, HS and data on the availability of essential medicine were used. For hospital access, we used annual in-patient admission or discharge per capita. For HWF density, we used the availability of health professionals: physicians, psychiatrists and surgeons per capita. For HS, we used the International Health Regulation core capacity index. Since this indicator is only available at the national level, it was excluded from the subnational analysis. For the measurement of essential medicines, we calculated the availability of the 14 WHO-recommended core list of essential medicines (ie, glibenclamide, beta-blocker, ACE inhibitor, simvastatin, amitriptyline, ciprofloxacin, co-trimoxazole, amoxicillin, ceftriaxone injection, diazepam tablet, diclofenac/ibuprofen, paracetamol and omeprazole) at health facilities:

$\begin{array}{l} HSCA=(Hospitalaccess*HWF*Essentialmedicines\\ {}*HSright{)}^{1/4} \end{array}$

Therefore, the overall UHC service coverage was computed within each of the four categories and then across those category-specific means to obtain the final summary index.^3^

$\begin{array}{l} UEHSC=(RMNCH*Infectious*NCD\\ {}*HealthServiceCapacityright{)}^{1/4} \end{array}$

We computed the regional-level UHC service coverage status in the same way as the national index and compared their distributions to the national coverage and among regions. In this analysis, the geometric mean was applied instead of the arithmetic mean because the geometric mean is less sensitive to extreme values.^19^

We tested the sensitivity of the index to see how the indicators were combined into a summary measure by recomputing the index, using the arithmetic means in addition to the geometric means that was performed in the base case. We also assessed the sensitivity of the index by dropping one indicator at a time: first, deleting the HS variable and then deleting the entire ‘health service and capacity’ component.

### Patient and public involvement statement

The study was approved by the Institutional Review Board (IRB) of the Ethiopian Public Health Institute (Ref: EPHI/6.13/607). No patient level data were used in this study.

## Results

**Figure 1:**
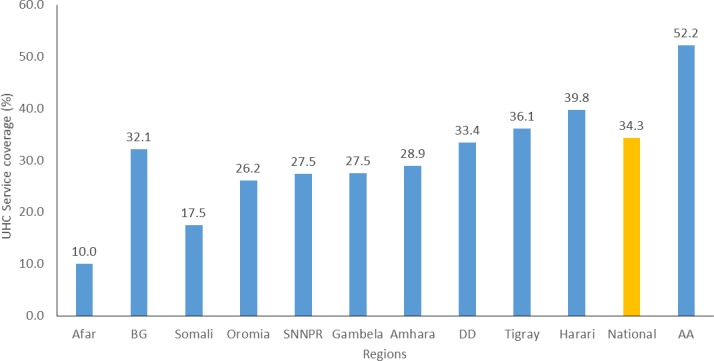
Overall UHC service coverage across regions. AA, Addis Ababa; BG, Benshangul Gumuze; DD, Dire Dawa; SNNPR, Southern Nations, and National Region; UHC, universal health coverage.

The Ethiopian overall UHC service coverage for the year 2015 was about 34.3% (table 2), ranging from 52.2% in Addis Ababa to 10% in Afar region (figure 1).

**Table 2:** Regional and national summary of UHC service coverage, 2015 Ethiopia

Tracer indicators	Tigray	Afar	Amhara	Oromia	Somali	BG	SNNPR	Gambela	Harari	AA	DD	National
RMNCH												
Family planning	35.2	11.6	46.9	28.1	1.4	28.4	39.6	34.9	29.3	50.1	29.1	35.3
Pregnancy care	57.9	18.4	29.5	20.9	15.4	34.7	33.1	45.1	42.3	92.9	61.2	29.8
Immunisation	81.4	20.1	63.8	39.9	36.3	76.2	59.0	54.8	58.7	95.7	84.9	53.2
Child treatment	34.1	41.3	31.4	35.0	26.8	41.6	36.7	45.0	53.8	62.5	51.2	35.3
UHC_RMNCH	48.8	20.5	40.8	30.1	12.0	42.0	41.0	44.4	44.5	72.6	52.7	37.5
Infectious diseases												
Tuberculosis treatment	59.6	59.6	59.6	59.6	59.6	59.6	59.6	59.6	59.6	59.6	59.6	59.6
HIV treatment	59.9	37.5	62.8	49.1	82.3	67.8	38.9	23.8	77.2	67.8	62.1	55.0
Malaria prevention	40.4	50.6	43.4	41.0	37.7	40.3	35.5	42.3	71.0	N/A	15.6	39.7
Improved water and sanitation	51.2	37.7	79.0	51.1	37.4	62.5	52.5	61.0	31.8	91.7	48.2	59.9
UHC_ID	52.1	45.4	59.9	49.8	51.3	56.5	45.6	43.7	56.8	71.8	40.8	52.8
Non-communicable diseases												
Treatment of CVD	70.8	58.5	55.6	61.6	69.0	62.0	48.4	66.3	62.6	40.7	75.2	58.2
Management of diabetes	100.0	100.0	93.6	100.0	74.4	100.0	100.0	100.0	100.0	100.0	100.0	100.0
Cervical cancer screening	1.2	0.0	1.6	1.4	8.0	1.5	1.0	2.0	0.8	11.0	3.7	2.9
Tobacco control	99.6	81.2	96.4	88.8	71.0	89.6	92.8	56.0	67.2	93.2	75.8	89.2
UHC_NCD	30.3	2.6	29.9	29.6	41.3	30.2	25.9	29.4	24.1	45.2	38.1	35.0
Service capacity and access												
Hospital access	26.1	4.5	9.4	13.4	2.3	15.3	10.9	23.7	81.8	35.7	44.3	13.1
Health worker density	8.0	0.4	1.6	1.7	0.4	5.9	2.8	1.4	19.7	15.0	1.6	3.1
Access to EM	51.2	41.2	57.6	52.4	54.2	36.4	52.8	31.1	43.2	58.8	49.8	50.8
Health security	Na	Na	Na	Na	Na	Na	Na	Na	Na	Na	Na	78.0
UHC_SCA	22.0	4.2	9.5	10.6	3.7	14.9	11.7	10.1	41.1	31.6	15.2	20.0
Overall UHC index	36.1	10.0	28.9	26.2	17.5	32.1	27.5	27.5	39.8	52.2	33.4	34.3

AA, Addis Ababa; BG, Benshangul Gumuze; CVD, cardiovascular disease; DD, Dire Dawa; NCDs, non-communicable diseases; RMNCH, reproductive, maternal, neonatal and child health; SCA, service capacity and access; SNNPR, Southern Nations, and National Region; UHC, universal health coverage.

**Figure 2:**
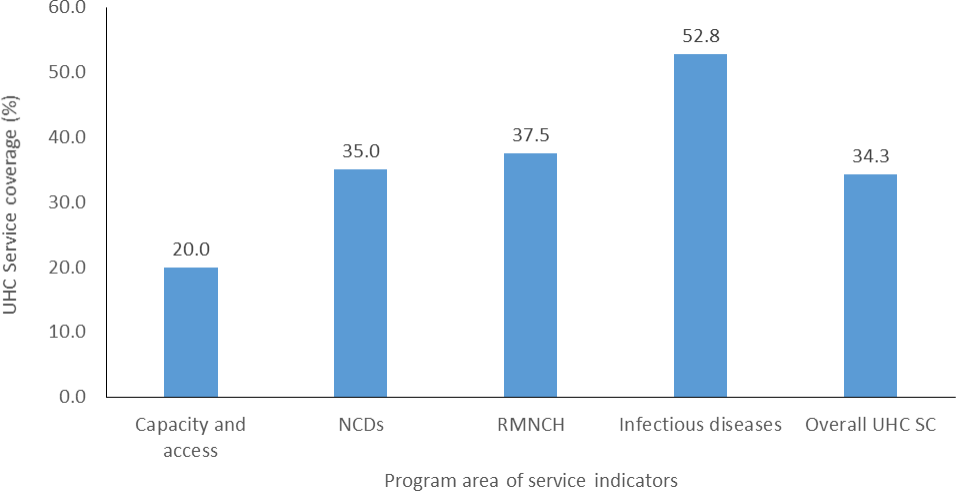
UHC service coverage across area of service indicators. NCD, non-communicable diseases; RMNCH, reproductive, maternal, neonatal and child health; UHC, universal health coverage.

We also explored UHC service coverage variation across areas of service indicators (figure 2). The national RMNCH service coverage was 37.5%; in this category, PC (29.8%) was the lowest performing area, as traced by FP (35%) and child treatment (35.3%). However, immunisation coverage was relatively higher (53.2%) in this category.

The national UHC service coverage for the infectious disease category was 52.8%. In this category, the lowest coverage was for LLIN (39.7%), followed by HIV treatment (55%) and TB treatment (59.6%). The highest coverage in this category was for improved water and sanitation (59.9%).

**Figure 3:**
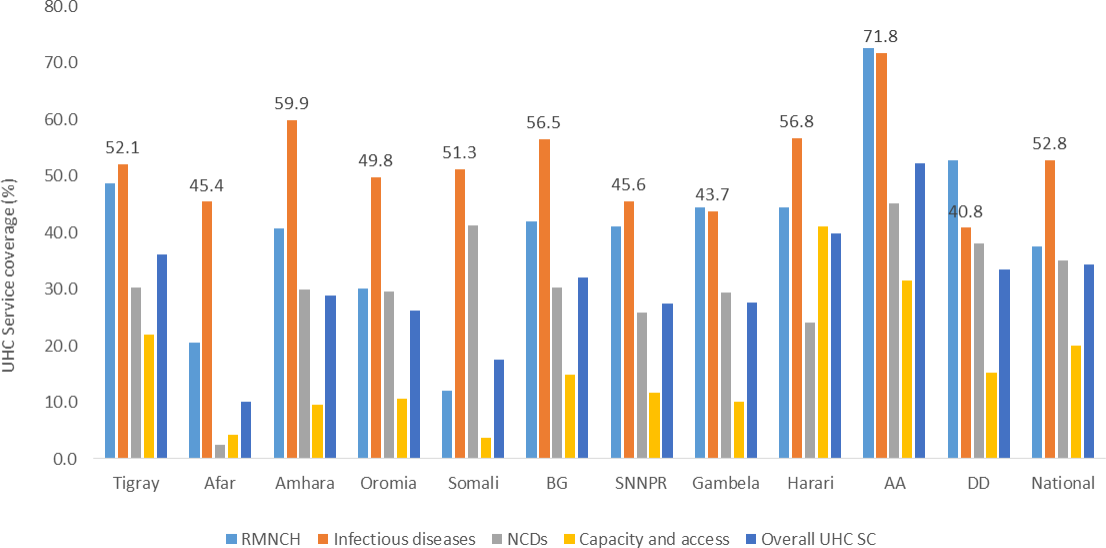
Regional variation of UHC SC tracer indicators. AA, Addis Ababa; BG, Benshangul Gumuze; DD, Dire Dawa; NCD, non-communicable diseases; RMNCH, reproductive, maternal, neonatal and child health; SNNPR, Southern Nations, and National Region; UHC, universal health coverage.

The national UHC for the NCD category was 35%, while the national UHC service capacity and access coverage was only 20%, with the highest variation across regions ranging from 3.7% in Somali to 41.1% in Harari (figure 3).

The UHC index was very sensitive to the choice of summary method. If we had chosen to use the arithmetic mean instead of the geometric mean, the UHC index would be 47.7%. Similarly, the index is sensitive to the type of indicators selected. Deleting the ‘health service and capacity’ component resulted in a UHC index of 41.1%, while deleting the HS variable would have resulted in a UHC index of 30.3%.

## Discussion

In this study, we attempted to estimate UHC service coverage for Ethiopia using 16 tracer indicators classified into four major groups: RMNCH, infectious disease, NCD and service access and capacity. We identified low UHC service coverage, substantial regional variations and a difference in achievement across programme areas. These findings trigger two key policy-relevant questions: Will Ethiopia achieve the UHC-SDG target, given the current level? How can we narrow the subnational variations and the large differences by programme areas?

### Low UHC service coverage

In this study, we identified that the 2015 overall UHC service coverage for Ethiopia (34.3%) was very low. This coverage level is substantially behind the SDG target of 80% by the year 2030 and also much lower compared with most Eastern African countries.^2^ For instance, the 2015 WHO/WB UHC service coverage estimate for Eastern African countries ranged from 39% in Tanzania to 57% in Kenya. A similar study by Barasa *et al* found a UHC service coverage for Kenya of about 42% in 2013.^14^ The coverage for Ethiopia is also considerably lower compared with the global average service coverage (64%) and sub-Saharan Africa average (42%).^3^

This low service coverage can be partially explained by demand-side factors, such as high multidimensional poverty among a large majority of the Ethiopian households.^23 24^ In Ethiopia, more than one-fourth of the entire population lives under the absolute poverty-line.^25 26^ Also, the low literacy rate, together with poor modern healthcare-seeking behaviour, could contribute to low coverage.^27^ From the supply side, minimal investment in Ethiopian health services could be the main reason for low UHC service coverage. For instance, although WHO recommends investing about US$112 per person annually (for low-income countries) to achieve the UHC-SDG target,^28^ Ethiopia was only investing about US$28 per capita.^29^ Therefore, it is reasonable to assume that the health infrastructure and human resources for health could have been better in Ethiopia if adequate resources had been mobilised in the health sector. However, achieving UHC requires the mobilisation of resources and equitable, transparent and efficient allocation and use of resources.^30^

Furthermore, our estimate (34.3%) is lower than an estimate by Hogan *et al*. (39%) and the WHO/WB report on tracking progress towards UHC for Ethiopia.^3 11^ This can be explained by the difference in the number of tracer indicators included and data sources applied in the studies. While our study includes all 16 recommended tracer indicators from more reliable data sources, Hogan *et al* used only 14 of the tracer-indictors (ie, excluding cervical cancer screening and access to essential medicine).

### Variation in achievement across programme area

As presented in detail in the WHO/WB joint technical report, the selection of the 16 tracer indicators is based on the definition of SDG 3.8.1, and all indicators are included with the intent to represent a given section of the health system.^11^ Countries should show progress in all areas of the tracer indicators to achieve UHC. Therefore, the comparison of tracer indicators across programme areas is important in identifying the gaps. Our study demonstrates immense variation in service coverage across different components of the UHC progress indicators. The national coverage for infectious disease (52.8%) is higher than RMNCH (37.5%) and NCDs (35%). The service capacity and access coverage (20%) was the lowest. The difference between higher coverage for infectious disease and service capacity and access might somehow reflect disease-oriented ‘priorities of the health system’ in previous years in Ethiopia as well as globally.^31^ This discrepancy was also reflected in the healthcare financing landscape that the larger share of the health resource in Ethiopia had been spent on major infectious diseases (ie, HIV, TB and malaria), followed by RMNCH services. The discrepancy can be partly ascribed to the influence of ‘donor-driven prioritisation’. For example, cervical cancer screening coverage is very low (about 3%). Therefore, the reprioritisation of health services—based on local disease burden data—in a way that can provide equitable and sustainable service provision may be an important consideration for the Ministry of Health to improve the gap across the UHC programme area and increase the total health gained for a fixed amount of the available budget.^32^

In terms of service capacity and access, most of the UHC coverage indices are low. Access to essential medicine was 50.8%, and only 13% of the population had access to hospital admission service. Alarmingly, we identified that there is a very low health worker density (3.1%). Since Ethiopia has invested in low-skilled health extension workers at community level (with 1–2 years of health education), the human resource profile in Ethiopia indicates that there is a shortage in many of the highly skilled healthcare provider categories recommended by global standards, and there is a critical shortage of surgeons and psychiatrists. For instance, there are only about 35 psychiatrists and 190 surgeons in the country for a population of 105 million. A study in central Ethiopia also reports a similar finding using hospital-based data.^33^ Therefore, the Ethiopian health system should clearly define a minimum set of important health services that are vital to the Ethiopian population and define the necessary workforce and health technologies, including essential medicine and physical infrastructure that matches the size and mix of the health needs of the whole population. Furthermore, the Ministry of Health should undertake a detailed analysis and close monitoring of the HWF density and distribution for all HWF categories both at national and subnational levels. Most importantly, these minimum inputs should be linked with adequate, sustainable financing mechanisms to ensure its continuity.

The relatively good coverage of tobacco control (89.2%), management of diabetes (100%) and CVD treatment (58.2%) can be due to the physically active lifestyle of the large majority of the Ethiopian population (ie, mostly rural residents and physically vigorous working conditions). However, the methodological limitations of how the indicators are constructed should be taken into account when interpreting these findings. In this study, tobacco control was defined as adults age ≥15 years not smoking tobacco in the last 30 days of the surveying period; management of diabetes is defined as the prevalence of non-raised blood glucose; and CVD treatment is defined as the prevalence of non-raised BP. Key components of the indicator are based on risk factors also influenced by factors other than service provision and access. To adjust for this limitation, we included coverage of cervical cancer screening. Without this indicator, the mean score for NCDs would have been much higher (and incorrect). Therefore, we recommend that these tracer indicators be replaced with indicators that directly measure the performance of the health system (if possible, effective coverage indicators). Similarly, a clear way of measuring the HS parameters should also be defined.

### Subnational variation

Our study reveals that there was huge subnational variation across different areas, ranging from service coverage of about 10% in the Afar region to 52.2% in the capital. Essentially, the variability in the UHC SC index might be partially explained by variations in both supply-side factors (ie, availability of health personnel and infrastructure) and demand-side factors (ie, socioeconomic status and literacy rate). The service coverage in the emerging regions, such as Afar, Benshangul Gumuze, Somali region and Gambla, was far lower than the national average. The target stated in the Health Sector Transformation Plan is to bring coverage of the emerging regions to the national average.^8^

Therefore, policymakers and programme managers in Ethiopia should give more attention to the needs of emerging regions. For instance, an intensified, targeted and focused intervention can be launched, special technical support can be organised and the pastoralist programme can be strengthened. In addition, the Federal Ministry of Health, together with RHBs of the emerging regions, should design a joint strategic plan to help bring regions with low UHC coverage to reach the national average in a short time. These strategies might include allocating additional funds from the national treasury for health in emerging regions; training and deployment of new highly skilled health professional to pastoralist regions that will also need incentives to retain such workforces; expansion of health infrastructure; improvement of procurement and distribution of essential drugs, supplies and medical equipment and designing region-specific implementation plans that take the regional context into account. Regional governments should also increase the proportion of allocated resource to the health-sector from the regional budget.

### Strength and limitations of the study

This study is the first of its kind in both fully constructing the UHC measurement index and measuring UHC service coverage at the national and subnational (regional) levels. However, the current study has some limitations related to either the selection of tracer indicator or the data sources, which require results to be interpreted with care. First, the identification of appropriate tracer indicators among several measures of service indicators is challenging for different reasons. In theory, the UHC encompasses several health service components. Second, the availability of appropriate data was a huge challenge and, therefore, some of the tracer indicators were proxy indicators, which only demonstrated the extent of the disease burden (eg, the prevalence of hypertension and FPG) rather than the actual service coverage.

Moreover, the coverage index does not adjust for quality. Findings from Service Provision Assessment (SPA) and SARA, as well as other studies, indicate that the quality of service provision is low and has substantial regional variation.^34 35^ Although data were not available to estimate effective coverage, as defined by Ng *et al*, it is not unreasonable to assume the effective coverage would be even lower than 34.3%.^36^

Regarding the data sources, we extensively applied survey data rather than routine administrative data. However, since survey data were not available to estimate service coverage of ART for HIV/AIDS, routine data from the health information management system was applied. Similarly, for tuberculosis, data from the WHO data repository were used. These routinely collected data might have limitation since these data were mainly collected by health professionals at health posts and health centres who have limited skill and a weak data quality monitoring system.^37 38^

Although wealth quintile data for some of the tracer indicators can be established from the survey data, disaggregated data by household socioeconomic status was lacking for some of the tracer indicators. Thus, we did not include inequality analysis across wealth quintiles.

## Conclusion

The overall UHC service coverage for Ethiopia in 2015 was 34.3%. That outcome is much lower compared with other countries in the region and the SDG-UHC target by 2030. There are also substantial subnational variations in coverage indicators and, therefore, an intensified effort is needed to achieve the intended result within the next 10 years nationally, especially in the regions lagging behind the national average. In addition, policymakers should be aware of the regional variation in UHC service coverage indicators and take corrective measures to narrow the gap across regions. The Ministry of Health should also start benchmarking progress towards UHC at the subnational level. In addition, a catch-up plan for regions that are lagging should be designed, such as redistribution of the HWF, increased resource allocations to health and focused technical and financial supports. In sum, Ethiopia should scale up its coverage of promotive, preventive and curative health service through increasing its investment in primary healthcare.
